# A controlled comparative study of the effects of methotrexate and pharmacogenetic factors on arterial blood pressure and arterial stiffness in patients with rheumatoid arthritis

**DOI:** 10.1080/07853890.2025.2539311

**Published:** 2025-07-31

**Authors:** Arduino A. Mangoni, Michael D. Wiese, Richard J. Woodman, Salvatore Sotgia, Angelo Zinellu, Ciriaco Carru, Julie-Ann Hulin, E. Michael Shanahan, Sara Tommasi

**Affiliations:** ^a^ College of Medicine and Public Health, Flinders Health and Medical Research Institute, Flinders University, Adelaide, Australia; ^b^ Department of Clinical Pharmacology, Finders Medical Centre, Southern Adelaide Local Health Network, Adelaide, Australia; ^c^ Centre for Pharmaceutical Innovation, Clinical & Health Sciences, University of South Australia, Adelaide, Australia; ^d^ Centre for Epidemiology and Biostatistics, College of Medicine and Public Health, Flinders University, Adelaide, Australia; ^e^ Department of Biomedical Sciences, University of Sassari, Sassari, Italy; ^f^ Medical Oncology Unit, University Hospital (AOUSS), Sassari, Italy; ^g^ Department of Rheumatology, Flinders Medical Centre, Southern Adelaide Local Health Network, Adelaide, Australia

**Keywords:** Methotrexate, inflammation, rheumatoid arthritis, drug repositioning, atherosclerosis, cardiovascular disease, blood pressure, pharmacogenetics

## Abstract

**Introduction:**

Observational studies have shown that methotrexate, a conventional synthetic disease-modifying antirheumatic drug (csDMARD), is associated with lower arterial blood pressure (BP) and may reduce cardiovascular risk in rheumatoid arthritis (RA). However, it remains unclear whether a cause-and-effect relationship exists between the use of methotrexate and blood pressure reduction.

**Patients and methods:**

We conducted a controlled comparative study of treatment-naïve newly diagnosed RA patients commenced on subcutaneous methotrexate (Group 1, *n* = 31, age 57 ± 15 years, 65% females, Disease Activity Score-28 – C-reactive protein, DAS28-CRP = 4.7 ± 1.2) or the DMARD comparator sulfasalazine (Group 2, *n* = 31, 54 ± 17 years, 61% females, DAS28-CRP = 5.0 ± 0.8). Clinic systolic (SBP, primary study endpoint) and diastolic BP (DBP) and augmentation index (AIx, a marker of arterial stiffness) were measured at baseline and after one and six months of treatment (ClinicalTrials.gov: NCT03254589).

**Results:**

After six months, compared to Group 2, Group 1 patients had significantly lower SBP (−7.4 mmHg, 95% CI −14.0 to −0.8, *p* = 0.03). By contrast, there were no significant between-group differences in DBP (*p* = 0.18), AIx (*p* = 0.85), or DAS28-CRP (*p* = 0.16). A significant effect of single nucleotide polymorphisms (SNPs) rs1801133 (methyl tetrahydrofolate reductase) and rs2231142 (ATP-binding cassette subfamily G member 2) on BP changes during methotrexate treatment was observed.

**Conclusions:**

This is the first comparative study showing that methotrexate significantly reduces SBP in RA. This effect did not coincide with significant changes in arterial stiffness or disease activity. Further research is warranted to investigate the mechanisms underpinning the SBP-lowering effects of methotrexate, the role of specific SNPs, and whether such effects may account for reduced cardiovascular risk in patients with RA.

## Introduction

Dysregulation of inflammatory pathways, leading to excessive local and systemic inflammation, is common in atherosclerosis and rheumatoid arthritis (RA) [1–4]. Studies have also reported vascular manifestations of atherosclerosis, including endothelial dysfunction [5–7], intima-media thickening [8], increased arterial stiffness [9], and a higher risk of arterial hypertension [10–12], in patients with RA. Elevations in arterial blood pressure and arterial stiffness, in turn, lead to increased cardiac afterload and energy demands, as well as an increased risk of coronary ischemia [13–16]. Not surprisingly, RA patients have a higher risk of incident cardiovascular events, e.g. myocardial infarction and stroke, when compared to the general population [17]. The increased cardiovascular risk in RA is likely due to excess inflammation, immune activation, and traditional risk factors (e.g. diabetes, arterial hypertension, and metabolic syndrome) that are overrepresented in the RA population [18,19].

The close interplay between inflammation and atherosclerosis has stimulated drug discovery programs to identify new therapies that exert atheroprotective effects by suppressing inflammation and immune activation [20–22]. Studies have also investigated the repurposing (repositioning) of marketed immunomodulatory and anti-inflammatory medications, such as disease-modifying antirheumatic drugs (DMARDs), as potential anti-atherosclerotic agents [23–25].

Systematic reviews and meta-analyses of observational studies have reported that treatment with methotrexate, a conventional synthetic DMARD (csDMARD) [26–28], is associated with significantly reduced cardiovascular risk in patients with RA and other autoimmune conditions compared to other DMARDs [29,30]. In these studies, methotrexate was associated with significantly lower all-cause mortality (hazard ratio, HR = 0.59, 95% CI 0.50 to 0.71, *p* < 0.001), cardiovascular mortality (HR = 0.72, 95% CI 0.53 to 0.97, *p* = 0.031), and cardiovascular morbidity (relative risk, RR = 0.80, 95% CI 0.73 to 0.88, *p* < 0.001). Such protective effects have not been observed with several other DMARDs [31–33].

Although studies have also assessed the effects of methotrexate on various cardiovascular risk factors, the most robust evidence has been derived from those investigating arterial blood pressure and hypertension [34]. For example, in observational studies in RA patients commencing different csDMARDs, methotrexate users experienced the most significant reductions in arterial blood pressure. They were also more likely to achieve optimal blood pressure control after six months [35]. Furthermore, in a repeated cross-sectional study conducted by our group, RA patients treated with methotrexate had significantly lower arterial blood pressure compared to patients treated with other csDMARDs, after adjusting for several confounders [36,37]. A reduced risk of hypertension in RA patients treated with methotrexate has also been reported in a systematic review [38]. However, the results of cross-sectional and observational studies do not allow a cause-and-effect relationship to be established between treatment with methotrexate and blood pressure lowering.

We sought to address this issue by investigating the temporal effects of methotrexate treatment on arterial blood pressure and arterial stiffness in patients with treatment-naïve, newly diagnosed RA. We also investigated the potential association between a range of single nucleotide polymorphisms (SNPs) regulating the effects of methotrexate and the changes in arterial blood pressure.

## Patients and methods

### Study population

This was an investigator-initiated study funded by medac GmbH (Germany) assessing a consecutive series of adult RA patients attending the Department of Rheumatology outpatient clinics within the Southern Adelaide Local Health Network, Adelaide, Australia (Trial registration number: NCT03254589) [39]. Supplementary Table S1 describes the inclusion, exclusion, and withdrawal criteria. The main inclusion criteria were adult treatment-naïve patients with a recent diagnosis of RA as defined by the European Alliance of Associations for Rheumatology (EULAR)/American College of Rheumatology (ACR) 2010 criteria [40,41]. The main exclusion criteria were contraindication to study treatment, severe, secondary, or resistant hypertension, significant hypotension, atrial fibrillation, heart failure, advanced chronic kidney disease, cancer, dementia, and uncontrolled diabetes or dyslipidaemia. The study was approved by the Southern Adelaide Clinical Human Research Committee (OFR # 26.17 – HREC/17/SAC/46). In accordance with the guidelines of the Declaration of Helsinki, all participants provided written informed consent before the study. We used the Consolidated Standards of Reporting Trials (CONSORT) guidelines [42].

### Intervention

This was a controlled comparative study in which RA patients were assigned to treatment with subcutaneous methotrexate (Group 1) or oral sulfasalazine, a csDMARD comparator, (Group 2) for six months. Patients in Group 1 were initially prescribed 10 mg of subcutaneous methotrexate once a week, and the dose was adjusted in 5 mg increments by the treating rheumatologist to a maximum 25 mg a week based on clinical response. Group 1 patients were also advised to take 5 mg of folic acid orally one to three days after the methotrexate dose to minimize the risk of adverse effects during methotrexate treatment [43]. Patients in Group 2 were initially prescribed 500 mg of sulfasalazine daily, and the daily dose was increased by 500 mg each week by the treating rheumatologist according to clinical response to a maximum dose of 3 g daily. The full blood count and liver function tests were monitored every four weeks for the first three months, then three monthly thereafter.

Assignment to Group 1 or Group 2 was guided by clinical evaluation by the treating rheumatologist and was non-blinded to both patients and rheumatologists to facilitate monitoring of response and management of side effects. The research team members not involved in patient management conducted study assessments and data collection. Patients and rheumatologists were asked not to disclose treatment details to these members.

A subcutaneous formulation of methotrexate, approved and registered in Australia for the treatment of RA, was selected for this study, given its higher bioavailability and reduced pharmacokinetic variability and incidence of gastrointestinal side effects compared to the oral formulation [44]. Sulfasalazine was chosen as a treatment comparator due to its relatively neutral effects on blood pressure and markers of atherosclerosis compared to other DMARDs [35,45].

### Study endpoints

The primary endpoint was the change in systolic blood pressure (SBP) during the treatment period. Secondary endpoints included changes in diastolic blood pressure (DBP), pulse pressure (PP), augmentation index (AIx, an indirect measure of arterial stiffness) [46], and disease activity (measured by the 28 joint Disease Activity Score with C-reactive protein, DAS28-CRP, and erythrocyte sedimentation rate, DAS28-ESR).

### Assessments

Each participant was assessed at baseline and then one and six months later. Data collected during each study visit included medical history, age (baseline only), sex (baseline only), weight, height, RA duration (baseline only), blood pressure, heart rate (HR), arterial stiffness, 28 tender and swollen joint counts, ESR, CRP, patient global health visual analog scale (VAS) (used to calculate DAS28-CRP and DAS28-ESR) [47–49], current medications, biochemical tests including full blood count and liver function tests, lifestyle assessment (diet and exercise), and adverse events.

#### Blood pressure and arterial stiffness

Blood pressure and HR were measured in the morning in a quiet environment at room temperature using an automated blood pressure monitor (Omron HEM-7230, Omron, Kyoto, Japan) [50]. According to current guidelines, three measurements were performed, and the average of the last two readings was used for analysis [50]. AIx, an indirect marker of arterial stiffness, was assessed using pulse wave analysis (SphygmoCor, AtCor Medical, West Ryde, NSW, Australia) [51]. Before assessing blood pressure, HR, and AIx, patients fasted for ≥12 hrs and abstained from alcohol for ≥12 hrs and from tobacco and caffeine for ≥4 hrs.

#### Genetic polymorphisms

We investigated the potential role of a range of SNPs shown to be important in the pharmacological effects of methotrexate in RA, or demonstrating a theoretical association with the cardiovascular effects of the drug.

#### Genomic DNA isolation

Genomic DNA was isolated from whole blood taken at baseline for each patient. Extractions were performed using the QIAamp DNA Blood Mini Kit (QIAGEN, Hilden, Germany; #51304) and a microcentrifuge, as per the manufacturer’s instructions. Genomic DNA was eluted in a final volume of 150 µL and sample concentrations were quantified using a NanoDrop 2000 (Thermo Fisher Scientific, Waltham, MA, USA). Samples were diluted to 4.5 ng/µL for genotyping and stored at −20 °C until time of analysis.

#### PCR amplification of the thymidylate synthase (TS) promoter 28 bp repeat

PCR reactions were carried out in 20 µL volumes using Phire Hot Start II DNA polymerase (Thermo Fisher Scientific, Waltham, MA, USA) according to the manufacturer’s instructions. Briefly, reactions comprised 2 µL genomic DNA (9 ng), 0.4 µL Phire, 4 µL 5x Phire buffer, 0.4 µL 10 nM dNTPs, primers at a final concentration of 0.5 µM, and water. The primer sequences were previously reported and were as follows [52,53]; Forward: 5′-GTG GCT CCT GCG TTT CCC CC-3′ and Reverse: 5′-GCT CCG AGC CGG CCA CAG GCA TGG CGC GG-3′. PCR amplification consisted of an initial denaturation at 98 °C for 30 s, followed by 30 cycles of 98 °C for 5 s, 62 °C for five seconds and 72 °C for ten seconds, and a final extension at 72 °C for one minute. The amplified DNA fragments were separated on a 3% agarose gel with SYBRSafe DNA gel stain (Thermo Fisher Scientific, Waltham, MA, USA), and PCR products containing triple repeats (248 bp) were distinguished from those containing double repeats (220 bp). Patients who were homozygous for the triple repeat (3 R/3R) displayed only the larger PCR product, those who were homozygous for the double repeat (2 R/2R) displayed only the smaller PCR product, and those who were heterozygous (2 R/3R) displayed both PCR products.

#### Quantitative real-time PCR amplification with TaqMan assays

Genotyping was performed for 12 single nucleotide polymorphisms (SNPs): ATP-binding cassette subfamily B member 1 (ABCB1; rs1045642), ATP-binding cassette subfamily C member 2 (ABCC2; rs2273697), ATP-binding cassette subfamily G member 2 (ABCG2; rs2231142), adenosine monophosphate deaminase (AMPD1; rs17602729), 5-aminoimidazole-4-carboxamide ribonucleotide formyltransferase (ATIC; rs2372536), methyl tetrahydrofolate reductase (MTHFR; rs1801131 and rs1801133), 5-methyltetrahydrofolate-homocysteine methyltransferase (MTR; rs1805087), 5-methyltetrahydrofolate-homocysteine methyltransferase reductase (MTRR; rs1801394), serine hydroxymethyltransferase 1 (SHMT; rs1979277), solute carrier family 19 member 1 (SLC19A1; rs1051266), and gamma-glutamyl hydrolase (GGH; rs719235).

Pre-designed allele-specific TaqMan SNP genotyping assays were purchased from Applied Biosystems (Thermo Fisher Scientific, Waltham, MA, USA) for all SNPs except SLC19A1 (rs1051266) which was custom-designed using the following reference sequence, with the SNP highlighted in bold:

5’CCGCCCTGGCACCCAGCGCCCACGTGCCTATTCCAGAAGCTGCTCCCTGCCCACCCACTGGCGGCCGCCCCCGCATCCCGGCGCCCACGTGCCTATTCCAGACGCTGCTCCCCGCCCACCCACCCACAGGCGGCCGCCCGGCACCCCGGCACCCACATGCCTGCTCCCGCGTGAAGTTCTTGTCGGGCCCCAGGAGGTAGGGGGTGATGAAGCTCTCCCCTGGCCGTATCTGCGCCATGAAGCCGTAGAAGCAAAGGTAGCACACGAGG**[T/C]**GCCGCCAGGACCGGAGCTCGGGGTCAGGCCCAGGTTCCACGGGCACCTGCTTCTCCACCGCTGGGCTGGAGGGCACCATCCTGCTCAGGCCACGTGCAGCTCCGGAGGGGACGAAGGTGACGCTGTGCCTGGAAGGAGGGGTGGAGTCAGGGCACCTTGGAAGATGGTCTGCAGGCCTCCCTACCCCGCAAAACGAGGCCCAGTGCCGGCCTCCTCGCCACTCAGGACCAAACGCCACCCTGGAGACGAATGCAGACCCCAGACCCATCCTCACCCCGTAGCAC3’

SNPMasker software (http://bioinfo.ebc.ee/snpmasker/) was used to identify SNPs with a global minor allele frequency of greater than 0.05% within the reference sequence [54]. These identified SNPs were masked by replacing with ‘N’ in the reference sequence. This sequence was supplied to Applied Biosystems for the development of the SLC19A1 (rs1051266; G80A) TaqMan SNP genotyping assay as follows:

5’CCGCCCTGGCACCCAG**N**GCCCACGTGCCTATTCCAGAAGCTGCTCCCTGCCCAC**N**CACTGGCGGCCGCCCCCGCATCCCGGCGCCCACGTGCCTATTCCAGACGCTGCTCCCCGCCCACCCACCCACAGGCGGCCGCCCGGCACCCCGGCACCCACATGCCTGCTCCCGCGTGAAGTTCTTGTCGGGCCCCAGGAGGTAGGGGGTGATGAAGCTCTCCCCTGGCCGTATCTGCGCCATGAAGCCGTAGAAGCAAAGGTAGCACACGAGG**[T/C]**GCCGCCAGGACCGGAGCTCGGGGTCAGGCCCAGGTTCCACGGGCACCTGCTTCTCCACCGCTGGGCTGGAGGGCACCATCCTGCTCAGGCCACGTGCAGCTCCGGAGGGGACGAAGGTGAC**N**CTGTGCCTGGAAGGAGGGGTGGAGTCAGGGCACCTTGGAAGATGGTCTGCAGGCCTCCCTACCCC**N**CAAAACGAGGCCCAGTGCCGGCCTCCTCGCCACTCAGGACCAAACGCCACCCTGGAGACGAATGCAGACCCCAGACCCATCCTCACCCCGTAGCAC3’

All quantitative real-time PCR (qRT-PCR) reactions were performed using 384-well plates in a QuantStudio 7 Pro (Thermo Fisher Scientific) using standard mode thermal cycling settings. Reactions consisted of 5 µL total volume containing 0.25 µL 20x TaqMan SNP genotyping assay, 2.5 µL 2x Taqman genotyping mastermix (Thermo Fisher Scientific, Waltham, MA, USA; #4371355) and 2.25 µL genomic DNA (10 ng). Plates were sealed with adhesive film and centrifuged at 2,000 rpm for two minutes before analysis. The qRT-PCR conditions for the majority of SNP genotyping assays were: initial incubation at 95 °C for ten minutes, followed by 40 cycles of 95 °C for 15 s and 60 °C for one minute. For drug metabolizing enzyme (DME) SNP genotyping assays (rs2231142, rs1045642, and rs2273697), amplification consisted of an initial incubation at 95 °C for ten minutes, followed by 50 cycles of 95 °C for 15 s and 60 °C for 90 s. For all assays, a pre- and post-read was taken at 60 °C for 30 s at the beginning and end of the reaction, respectively. To determine patient sample genotype for each SNP, data was analysed and extracted using Design & Analysis Software 2.8.0 (Applied Biosystems, Thermo Fisher Scientific, Waltham, MA, USA). For each SNP, patients were designated as either homozygous for the reference allele (R/R), homozygous for the alternate allele (a/a) or heterozygous (R/a). Genotypic and allelic frequencies were calculated for the study population and compared to global frequencies provided by the Allele Frequency Aggregator (ALFA) project and available *via* NCBI database of SNPs (ncbi.nlm.nih.gov/snp) [55].

### Sample size calculation

Based on an observed mean difference of 6 mmHg (SD 12) in SBP between patients with RA treated with methotrexate vs. other DMARDs in our previous repeated cross-sectional analysis [36], a two-sided type 1 error rate = 0.05, one baseline measure, two post-baseline measures, and a correlation between measures of *r* = 0.6, 28 patients were required in each group. Accounting for a 10% attrition rate, 31 patients were therefore required in each group.

### Statistical analysis

The normality of the data was assessed using Q-Q normality plots, Kolmogorov-Smirnov tests, and histograms. Statistical analysis was performed using linear mixed models, which allowed for repeated observations of the same individuals over time. The independent variables in each model included group, time, and group X time as fixed effects, and the subject ID as a random effect. The estimated treatment effect was taken to be the coefficient for the group X time interaction, which allows adjustment for baseline values of the outcome. The mixed-effects modelling was performed using SAS software (version 9.4 M8, Cary, NC, USA). As a sensitivity analysis, to account for missing data, we also repeated the analysis using a mixed-effects model with 10 multiply imputed datasets created using the fully conditional specification with predictive mean matching (eight nearest neighbours). Variables included in the imputation were age, body mass index, gender, and SBP and DBP at baseline, one and six months. All hypotheses were tested using a two-sided type 1 error rate of alpha = 0.05. Analysis of non-repeated measures data was performed using Stata (version 18.0, College Station, TX, USA).

Pharmacogenetic analyses were performed using IBM SPSS Statistics (V 29.0.1.0 (171)). Data from treatment Groups 1 and 2 were combined for assessment. To gain statistical power, assessments were made using a dominant model (RR vs. Ra + aa) and a recessive model (RR + Ra vs. aa), rather than an additive model (RR vs. Ra vs. aa). For each SNP, two-way analysis of covariance (ANCOVA) with multiple pairwise comparisons was performed to assess differences in the change in SBP, DBP, PP, or DAS28-CRP between treatment group and genotype over one and six months. Age, sex, and baseline values for the dependent variable were controlled for as covariates. P-values were corrected for multiple comparisons using the Bonferroni method and were considered statistically significant when *p* < 0.05. Data are presented as estimated means ± standard error.

## Results

### Clinical and demographic characteristics

The study recruitment started in 2017 and ended in 2023. The baseline characteristics of patients in Group 1 (methotrexate, *n* = 31) and Group 2 (sulfasalazine, *n* = 31) are described in Table 1. There were no significant between-group differences in demographic and clinical characteristics, particularly age, sex, RA duration, DAS28-CRP and DAS28-ESR, history of hypertension, and use of antihypertensive medications, corticosteroids, and non-steroidal anti-inflammatory drugs. Baseline SBP, DBP, PP, HR, and AIx values were also similar between the two groups. The allelic frequencies of assessed SNPs in Group 1 and Group 2, described in Supplementary Tables S2 and S3, were both highly concordant with the global frequencies.

**Table 1.** Baseline characteristics of group 1 (methotrexate) and group 2 (sulfasalazine).

	Group 1 (*n* = 31)	Group 2 (*n* = 31)	p-value
Age (years)	57 ± 15	54 ± 17	ns
Females (%)	65	61	ns
BMI (Kg/m^2^)	30 ± 6	31 ± 8	ns
RA duration (months)	22 ± 64	5 ± 14	ns
Patient global health VAS (mm)	50 ± 23	56 ± 23	ns
DAS28-CRP	4.73 ± 1.20	5.00 ± 0.83	ns
DAS28-ESR	5.29 ± 1.44	5.31 ± 1.22	ns
CRP (mg/L)	4.0 ± 3.6	7.0 ± 7.4	ns
ESR (mm/hr)	24 ± 17	24 ± 21	ns
Alcohol use (%)	71	58	ns
Diabetes (%)	19	16	ns
Hypertension (%)	36	19	ns
Hypercholesterolaemia (%)	36	36	ns
Chronic kidney disease (%)	0	3	ns
Myocardial infarction (%)	3	3	ns
Stroke (%)	0	6	ns
Obesity (%)	32	39	ns
COPD (%)	3	16	ns
Cigarette smoking (%)	13	19	ns
Aspirin (%)	10	10	ns
Antihypertensives (%)	35	19	ns
Corticosteroids (%)	45	39	ns
NSAIDs (%)	19	32	ns
SBP (mmHg)	127 ± 16	122 ± 19	ns
DBP (mmHg)	83 ± 9	81 ± 9	ns
PP (mmHg)	44 ± 14	41 ± 16	ns
HR (bpm)	70 ± 12	70 ± 10	ns
AIx (%)	23 ± 9	21 ± 13	ns

Notes: BMI, body mass index; RA, rheumatoid arthritis; VAS, visual analog scale; DAS28, Disease Activity Score-28; CRP, C-reactive protein; ESR, erythrocyte sedimentation rate; COPD, chronic obstructive pulmonary disease; NSAIDs, non-steroidal anti-inflammatory drugs; SBP, systolic blood pressure; DBP, diastolic blood pressure; PP, pulse pressure; HR, heart rate; AIx, augmentation index.

### Follow-up

In Group 1, 30 participants completed the follow-up at one month and 26 at six months. The reasons for study withdrawal in Group 1 participants (*n* = 5) included adverse events (*n* = 2), switching to alternative treatment (*n* = 1), failure to attend or comply (*n* = 1), and being no longer eligible (*n* = 1). In Group 2, 26 patients completed the follow-up at one month and 15 at six months. The reasons for study withdrawal in Group 2 participants (*n* = 16) included adverse events (*n* = 5), switching to alternative treatment (*n* = 6), personal reasons (*n* = 2), failure to attend or comply (*n* = 2), and being no longer eligible (*n* = 1).

There were no significant between-group differences at six months in the use of non-steroidal anti-inflammatory drugs (3% vs. 11%, *p* = 0.130), corticosteroids (15% vs. 7%, *p* = 0.636), or use of antihypertensive medications (31% vs. 20%, *p* = 0.716).

### Adverse events

Non-severe adverse events in Group 1 included atrial fibrillation (*n* = 1), abnormal liver function (*n* = 1), gastrointestinal symptoms (*n* = 2), hair loss/thinning (*n* = 2), feeling of excessive heat (*n* = 2), dizziness (*n* = 1), fatigue (*n* = 1), mood change (*n* = 1), memory impairment (*n* = 1), mouth ulcers (*n* = 1), face swelling (*n* = 1), and appetite loss (*n* = 1). Non-severe adverse events in Group 2 included nausea (*n* = 4), loss of appetite (*n* = 1), nightmares (*n* = 1), night sweats (*n* = 1), fever (*n* = 1), gastrointestinal symptoms (*n* = 1), dizziness (*n* = 2), tachycardia (*n* = 1), fatigue (*n* = 2), headache (*n* = 1), itchy skin (*n* = 1), and neutropenia (*n* = 1). None of these adverse events led to study withdrawal.

### Changes in blood pressure and arterial stiffness

The between-group differences in blood pressure and arterial stiffness at one and six months are presented in Table 2. At one month, Group 1 showed a trend towards significantly lower SBP values (*p* = 0.08) compared to Group 2, and there were no significant between-group differences in DBP, PP, or AIx. At six months, Group 1 had significantly lower SBP values when compared to Group 2 (*p* = 0.03), but, by contrast, there were no significant between-group differences in DBP, PP, or AIx.

**Table 2.** Between-group differences in blood pressure, augmentation index, and disease activity at one and six months.

Assessment	Variable	Δ (95% CI)	p-value
1-month follow up	SBP	−4.9 (−10.6, 0.7)	0.08
	DBP	−3.1 (−7.1, 1.0)	0.14
	PP	−1.9 (−6.2, 2.4)	0.38
	AIx	−1.1 (−4.2, 2.0)	0.48
	DAS28-CRP	−0.67 (−1.24, −0.10)	**0.02**
	DAS28-ESR	−0.46 (−1.15, 0.24)	0.19
6-months follow up	SBP	−7.4 (−14.0, −0.8)	**0.03**
	DBP	−3.0 (−7.5, 1.4)	0.18
	PP	−4.3 (−9.8, 1.1)	0.11
	AIx	0.3 (−2.8, 3.4)	0.85
	DAS28-CRP	−0.54 (−1.31, 0.23)	0.16
	DAS28-ESR	−0.6 (−1.32, 0.59)	0.44

Notes: SBP, systolic blood pressure; DBP, diastolic blood pressure; PP, pulse pressure; AIx, augmentation index; DAS28, Disease Activity Score-28; CRP, C-reactive protein; ESR, erythrocyte sedimentation rate. Bold values are highlighted for Significant differences.

**Figure 1.**
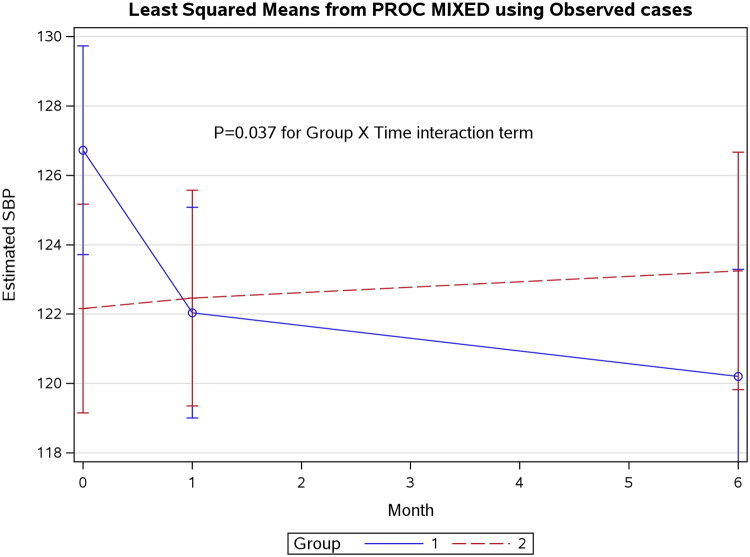
Effects of methotrexate (Group 1) and sulfasalazine (Group 2) on systolic blood pressure using a repeated measures mixed effects model.

**Figure 2.**
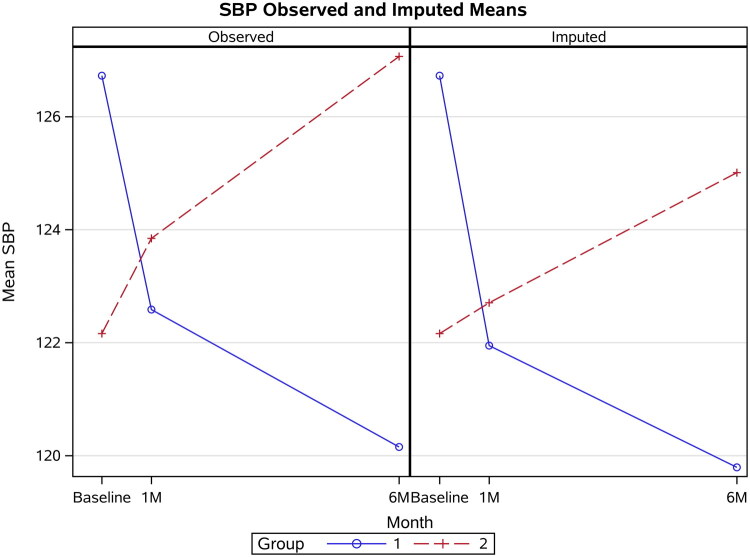
Observed and imputed means for systolic blood pressure (SBP) for group 1 (methotrexate) and group 2 (sulfasalazine). Observed means are based on observed cases only (*n* = 31/31 for group 1 and 2 at baseline; *n* = 29/26 for group 1 and 2 at one month; *n* = 26/15 for group 1 and 2 at six months). Imputed means are the means across *n* = 10 multiply imputed datasets with *N* = 31/31 for group 1 and 2 at baseline, one and six months.

Using the observed data only, the repeated-measures mixed-effects model revealed a significant group x month interaction for SBP (*p* = 0.037; Figure 1). The results were also significant when using the multiply imputed data (*p* = 0.026). For the observed data, the baseline-adjusted Group 2 vs. Group 1 interaction was significant at six months (β = 7.61 mmHg, 95% CI 1.53 to 13.67, *p* = 0.0159) but not at one month (β = 4.98 mmHg, 95% CI −0.29 to 10.26, *p* = 0.067). The baseline-adjusted SBP change with the 10 multiple imputation datasets showed a significant between-group difference (β = 9.78 mmHg, 95% CI 2.40 to 17.17, *p* = 0.0098) at six months (Figure 2) but no significant differences at one month (β = 5.32 mmHg, 95% CI −1.41 to 12.05, *p* = 0.121).

### Changes in disease activity

The between-group differences in DAS28-CRP and DAS28-ESR at one month and six months are illustrated in Table 2. At one month, Group 1 had a significantly lower DAS28-CRP value compared to Group 2 (*p* = 0.02). By contrast, there were no significant between-group differences in DAS28-ESR. At six months, there were no significant between-group differences in either DAS28-CRP or DAS28-ESR.

### Single nucleotide polymorphisms (SNPs)

Significant interactions between specific genetic polymorphisms within the methotrexate treatment group and changes in blood pressure and disease activity using ANCOVA were identified and are described in Table 3. At one month, individuals who carried one or more A alleles within MTHFR (rs1801133) had a greater reduction in SBP (*p* = 0.026) and DBP (*p* = 0.04), and those carrying one or more G alleles at ABCG2 (rs2231142) had a greater reduction in SBP (*p* = 0.049). After six months of methotrexate treatment, those who carried one or more T allele at ABCG2 (rs2231142) were more likely to have higher DBP (*p* = 0.046), and those who carried a G allele at MTRR (rs1801394) were more likely to have a lower DAS28-CRP (*p* = 0.028).

**Table 3.** ANCOVA analysis of genetic polymorphisms in interaction with methotrexate treatment on change in blood pressure and disease activity at one- and six-months follow-up.

1-month methotrexate (Group 1)									
	rs1801131 (MTHFR)			rs1801133 (MTHFR)			rs2231142 (ABCG2)		
	TT	TG+GG	p-value	GG	GA+AA	p-value	GG+GT	TT	p-value
SBP	−9.03 ± 2.59	−0.63 ± 2.49	0.025*	−0.96 ± 2.37	−9.17 ± 2.62	**0.026**	−5.23 ± 1.81	14.7 ± 9.75	**0.049**
DBP	−6.03 ± 1.81	−0.59 ± 1.71	0.034*	−0.71 ± 1.67	−6.01 ± 1.87	**0.04**	−3.18 ± 1.29	1.75 ± 6.84	0.482
PP	−2.73 ± 2.05	−0.14 ± 2.02	0.377	−0.23 ± 1.94	−2.81 ± 2.11	0.374	−1.89 ± 1.4	13.77 ± 7.67	0.049*
DAS28-CRP	−1.24 ± 0.29	−1.22 ± 0.27	0.959	−1.33 ± 0.26	−1.09 ± 0.3	0.547	−1.23 ± 0.2	−1.15 ± 1.04	0.947
6-month methotrexate (Group 1)									
	rs2231142 (ABCG2)			rs1801394 (MTRR)					
	GG	GT+TT	p-value	AA	AG+GG	p-value			
SBP	−7.08 ± 2.13	−4.95 ± 4.49	0.675	−9.28 ± 4.97	−6.19 ± 2.05	0.574			
DBP	−2.70 ± 1.45	4.43 ± 2.99	**0.046**	−0.17 ± 3.63	−1.48 ± 1.51	0.746			
PP	−4.49 ± 1.67	−9.05 ± 3.48	0.249	−9.01 ± 3.88	−4.69 ± 1.62	0.318			
DAS28-CRP	−1.61 ± 0.25	−0.9 ± 0.53	0.239	−0.25 ± 0.57	−1.71 ± 0.24	**0.028**			

Notes: SBP, systolic blood pressure; DBP, diastolic blood pressure; PP, pulse pressure; DAS28-CRP, disease activity score-28 – C-reactive protein, MTHFR, methyl tetrahydrofolate reductase; ABCG2, ATP-binding cassette subfamily G member 2; MTRR, 5-methyltetrahydrofolate-homocysteine methyltransferase reductase.

Values are estimated means ± SE. All adjusted for age, sex and baseline measurement of dependent variable. SBP, DBP and PP are expressed as change in mmHg.

*: Not significant at ANCOVA level, only at pairwise comparison. Bold values are highlighted for Significant differences.

Supplementary Table S4 describes significant interactions between genetic polymorphism and sulfasalazine treatment on changes in blood pressure and disease activity as determined by ANCOVA. At one month, individuals who carried the TT genotype at SLC19A1 (rs1051266) had a greater reduction in SBP (*p* = 0.003), those who carried the AA genotype at ABCB1 (rs1045642) had higher PP (*p* = 0.039), and those who carried a C allele at ATIC (rs2372536) had significantly lower DBP (*p* = 0.04). At six months, those who carried one or more G alleles at MTHFR (rs1801131) had significantly lower SBP (*p* = 0.027), those who carried a CC genotype at GGH (rs719235) had significantly lower DBP (*p* = 0.047), and those with TT at SLC19A1 (rs1051266) also had significantly lower DBP (*p* = 0.02).

## Discussion

In this study, six-month treatment with methotrexate significantly reduced the primary endpoint, SBP, when compared to sulfasalazine in a cohort of adult treatment naïve patients with newly diagnosed RA. The significant between-group differences in SBP were also observed using mixed models and multiple imputation analysis, accounting for missing data. Notably, the SBP-lowering effects of methotrexate did not coincide with significant changes in either measures of arterial stiffness (AIx) or disease activity. This suggests that the blood pressure-lowering effects of methotrexate are unlikely to be attributed to changes in arterial tone, arterial elasticity, or overall severity of RA.

The absence of effects of methotrexate on AIx is in line with the results of a repeated cross-sectional study previously conducted by our group comparing RA patients receiving treatment with methotrexate and other DMARDs (*n* = 56, age 61 ± 13 years, 70% females) and RA patients treated with DMARDs other than methotrexate (*n* = 30, age 63 ± 12 years, 76% females) [36]. The reported between-group differences in AIx (+0.6%, 95% CI −1.8 to 3.0, *p* = 0.65) were similar to those observed in the current study (+0.3%, 95% CI −2.8 to 3.4, *p* = 0.85). The lack of significant between-group changes in DAS28-CRP at six months corroborates the results of a recent observational study reporting a significant reduction in a composite of incident coronary artery disease, stroke, heart failure-related hospitalization, or cardiovascular mortality (HR = 0.76, 95% CI 0.58 to 0.99, *p* = 0.04) in 2044 RA patients treated with methotrexate (mean age 63.9 years, 90% males, baseline DAS28-CRP 3.6) [56]. In this study, there was no evidence of an indirect effect of methotrexate on cardiovascular risk through disease activity modification in mediation analyses (HR 1.03, 95% CI 0.80 to 1.32). Further research is warranted to investigate the mechanisms underpinning the methotrexate-associated reductions in SBP in RA, e.g. improved endothelial function and nitric oxide synthesis, reduced expression of specific pro-inflammatory cytokines, and changes in sympathovagal balance, as well as the effect of treatment on the structure and function of large and small arteries, given their specific involvement in the modulation of blood pressure [57].

Pharmacogenetic analysis revealed significant associations between rs1801133 (MTHFR) and rs2231142 (ABCG2) and SBP changes and DBP changes at one month and between rs2231142 (ABCG2) and DBP changes at six months. These results suggest the potential biological and clinical relevance of these SNPs for developing precision medicine interventions to optimize the blood pressure-lowering effects of methotrexate in RA. This proposition is supported by studies investigating the association between rs1801133 (MTHFR) and hypertension. A systematic review and meta-analysis of 49 studies investigating 9,613 patients with essential hypertension and 10,713 matched controls reported a significant association between the rs1801133 (MTHFR) polymorphism and the risk of hypertension (A-allele vs. G-allele, OR = 1.38, 95% CI 1.25 to 1.54, *p* < 0.001) [58]. Notably, in our study, RA patients with the A-allele (i.e. AA+AG) had more significant methotrexate-associated reductions in SBP and DBP at one month when compared to patients with the GG-genotype (−9.17 ± 2.62 vs. −0.96 ± 2.37 mmHg, *p* = 0.026; −6.01 ± 1.87 vs. −0.71 ± 1.67, *p* = 0.04, respectively) (Table 3). Furthermore, rs2231142 (ABCG2) has shown significant associations with elevated uric acid concentrations and gout, factors that have been traditionally associated with an increased risk of hypertension [59,60]. In our study, the methotrexate-associated reduction in SBP at one month was more significant in RA patients with the G-allele (GG+GT) when compared to those with the T-allele (TT, −5.23 ± 1.81 vs. +14.7 ± 9.75 mmHg, *p* = 0.049), and that of DBP at six months was more significant in the GG group when compared to the GT+TT group (−2.70 ± 1.45 vs. +4.43 ± 2.99 mmHg, *p* = 0.046). The specific associations between rs1801133 (MTHFR) and rs2231142 (ABCG2) and blood pressure changes during methotrexate treatment are supported by the concomitant lack of associations between these SNPs and blood pressure changes with sulfasalazine described in Supplementary Table S4.

The observed mean difference in SBP changes at six months between treatment with methotrexate and sulfasalazine, −7.4 mmHg, is similar (−6.0 mmHg) to that observed in our previous repeated cross-sectional study comparing RA patients receiving treatment with methotrexate and other DMARDs with RA patients treated with other DMARDs but not methotrexate [36]. This effect magnitude is biologically and clinically relevant as a 5-mmHg reduction in SBP following pharmacological treatment in landmark cardiovascular prevention trials has been shown to translate into a 10% reduction in the risk of developing major cardiovascular events in large individual participant-level data meta-analyses (HR 0.90, 95% CI 0.88 to 0.82) [61]. While additional investigations are required to confirm our findings in RA and non-RA populations, the presence of significant excess inflammation may also play an important role in the blood pressure responses to methotrexate. In a secondary analysis of the Cardiovascular Inflammation Reduction Trial (CIRT) investigating patients with high cardiovascular risk but without RA or other autoimmune disorders, treatment with low-dose methotrexate (*n* = 2391) did not significantly reduce SBP or DBP when compared to placebo (*n* = 2395) during a mean follow-up of 26 months (SBP: −0.75 mmHg, 95% CI −0.02 to −1.49; DBP: −0.56 mmHg, 95% CI −0.12 to −1.01) [62]. However, the median baseline CRP concentrations in CIRT participants (methotrexate group: 1.53 mg/L; placebo group: 1.50 mg/L) were considerably lower than in our study (methotrexate group: 4.0 ± 3.6 mg/L; sulfasalazine group: 7.0 ± 7.4 mg/L). Further studies should also investigate the association between baseline inflammatory burden and blood pressure responses to methotrexate in patients with RA, other autoimmune disorders, and patients without autoimmune disorders and a wide range of CRP concentrations. It is also worth noting that participants in the CIRT study received folic acid treatment both in the methotrexate and in the placebo group and that methotrexate was administered orally, with potentially higher pharmacokinetic variability compared to subcutaneous administration in our study [62]. Treatment with this B-vitamin has been shown to exert beneficial effects on endothelial function and cardiovascular risk in other studies, potentially mitigating the effects of methotrexate on blood pressure [63,64]. The concomitant use of folic acid and methotrexate in Group 1 in our study might have also contributed to the observed reduction in SBP. However, in a recent systematic review and meta-analysis of 22 randomized controlled studies, the magnitude of the reduction in SBP with folic acid treatment (weighted mean difference, WMD=-1.10 mmHg, 95%CI −1.93 to −0.28, *p* = 0.008) was relatively small when compared to mean SBP differences at six months (−7.4 mmHg) between methotrexate and sulfasalazine [65]. This suggests that methotrexate treatment was primarily responsible for the SBP-lowering effects in our study. This proposition is further supported by the lack of between-group differences in the use of other drugs affecting blood pressure, e.g. antihypertensive medications, corticosteroids, and non-steroidal anti-inflammatory drugs [66,67].

Patients with RA are commonly prescribed multiple medications for the management of the primary condition as well as specific comorbidities. Recent studies have reported a prevalence of polypharmacy, defined as the use of five or more concomitant medications in an individual patient, between 60% and 70% in this group [68,69]. The main classes of medications prescribed in these studies, in addition to corticosteroids, non-steroidal anti-inflammatory drugs, and DMARDs, included antihypertensives, lipid-lowering drugs, drugs for osteoporosis, hypoglycemics, antianemics, and medications for acid-related disorders. Given this complexity, further studies are required to investigate whether the blood pressure-lowering effects of methotrexate are influenced by the concomitant treatment with other drugs in the medium and long term.

Strengths of our study include a comprehensive assessment of blood pressure, arterial stiffness, measures of RA activity, and a wide range of SNPs potentially influencing the pharmacological effects of methotrexate, as well as hypertension and cardiovascular risk.

A significant limitation of the study is the treatment assignment to methotrexate or sulfasalazine based on clinical evaluation by the treating rheumatologist, rather than standard randomization, which introduces the potential for confounding by clinical indication due to selection bias. However, this potential bias was minimized by blinding the research team responsible for conducting the assessments and analysing the data from the treatment, using standard protocols for data collection and analysis to minimize inter-observer variability and improve consistency, clearly defining the inclusion and exclusion criteria, transparently reporting all results, and regularly auditing and monitoring the study. Most importantly, Table 1 demonstrates that the groups were very similar at the start of the study. Specifically, there were no significant differences in the primary measures of RA severity, the DAS28-CRP and the DAS28-ESR, suggesting that, on average, clinicians did not assign one drug to significantly ‘sicker’ patients. The groups were also similar in terms of age, sex, body mass index, and rates of comorbidities, such as diabetes and hypertension. Furthermore, the starting SBP, the primary endpoint, was not significantly different between the two groups. Another limitation is the relatively high dropout rate in patients receiving treatment with sulfasalazine. However, multiple imputation analysis, which accounted for missing data, confirmed the significant between-group differences in SBP changes at six months.

## Conclusion

Our study provides the first evidence that methotrexate treatment significantly reduces SBP over six months when compared to the DMARD sulfasalazine in treatment naïve, newly diagnosed patients with RA. Whilst the SBP reduction with methotrexate did not coincide with significant changes in arterial stiffness or disease activity, pharmacogenetic analysis showed the potential role of specific SNPs, rs1801133 (MTHFR) and rs2231142 (ABCG2), in influencing the blood pressure responses. Further research is warranted to identify the mechanisms underlying the SBP-lowering effects of methotrexate and whether such effects may account for the potential beneficial effects of the drug on cardiovascular risk in RA and non-RA populations.

## Supplementary Material

CONSORT_flow_diagram.doc

Supplementary_Tables.docx

CONSORT_checklist.docx

## Data Availability

The datasets used and/or analysed during the current study are available from the corresponding author on reasonable request.

## References

[1] Libby P, Buring JE, Badimon L, et al. Atherosclerosis. Nat Rev Dis Primers. 2019;5(1):56. doi: 10.1038/s41572-019-0106-z. 31420554

[2] Wolf D, Ley K. Immunity and inflammation in atherosclerosis. Circ Res. 2019;124(2):315–327. doi: 10.1161/CIRCRESAHA.118.313591. 30653442 PMC6342482

[3] Roy P, Orecchioni M, Ley K. How the immune system shapes atherosclerosis: roles of innate and adaptive immunity. Nat Rev Immunol. 2022;22(4):251–265. doi: 10.1038/s41577-021-00584-1. 34389841 PMC10111155

[4] Di Matteo A, Bathon JM, Emery P. Rheumatoid arthritis. Lancet. 2023;402(10416):2019–2033. doi: 10.1016/S0140-6736(23)01525-8. 38240831

[5] Erre GL, Piga M, Fedele AL, et al. Prevalence and determinants of peripheral microvascular endothelial dysfunction in rheumatoid arthritis patients: a multicenter cross-sectional study. Mediators Inflamm. 2018;2018:6548715–6548718. doi: 10.1155/2018/6548715. 29483841 PMC5816852

[6] Erre GL, Mangoni AA, Passiu G, et al. Comprehensive arginine metabolomics and peripheral vasodilatory capacity in rheumatoid arthritis: a monocentric cross-sectional study. Microvasc Res. 2020;131:104038. doi: 10.1016/j.mvr.2020.104038. 32622695

[7] Mangoni AA, Tommasi S, Sotgia S, et al. Asymmetric dimethylarginine: a key player in the pathophysiology of endothelial dysfunction, vascular inflammation and atherosclerosis in rheumatoid arthritis? Curr Pharm Des. 2021;27(18):2131–2140. doi: 10.2174/1381612827666210106144247. 33413061

[8] van Sijl AM, Peters MJ, Knol DK, et al. Carotid intima media thickness in rheumatoid arthritis as compared to control subjects: a meta-analysis. Semin Arthritis Rheum. 2011;40(5):389–397. doi: 10.1016/j.semarthrit.2010.06.006. 20889191

[9] Wang P, Huang L, Xu Q, et al. Assessment of aortic stiffness in patients with rheumatoid arthritis using pulse wave velocity: an update meta-analysis. Arch Med Res. 2019;50(7):401–412. doi: 10.1016/j.arcmed.2019.10.010. 31760330

[10] Panoulas VF, Douglas KM, Milionis HJ, et al. Prevalence and associations of hypertension and its control in patients with rheumatoid arthritis. Rheumatology (Oxford). 2007;46(9):1477–1482. doi: 10.1093/rheumatology/kem169. 17704521

[11] Panoulas VF, Metsios GS, Pace AV, et al. Hypertension in rheumatoid arthritis. Rheumatology (Oxford). 2008;47(9):1286–1298. doi: 10.1093/rheumatology/ken159. 18467370

[12] Liang X, Chou OHI, Cheung CL, et al. Is hypertension associated with arthritis? The United States national health and nutrition examination survey 1999-2018. Ann Med. 2022;54(1):1767–1775. doi: 10.1080/07853890.2022.2089911. 35786117 PMC9258429

[13] Milan A, Tosello F, Fabbri A, et al. Arterial stiffness: from physiology to clinical implications. High Blood Press Cardiovasc Prev. 2011;18(1):1–12. doi: 10.2165/11588020-000000000-00000. 21612307

[14] Boutouyrie P, Chowienczyk P, Humphrey JD, et al. Arterial stiffness and cardiovascular risk in hypertension. Circ Res. 2021;128(7):864–886. doi: 10.1161/CIRCRESAHA.121.318061. 33793325

[15] Chirinos JA, Segers P, Hughes T, et al. Large-artery stiffness in health and disease: JACC state-of-the-art review. J Am Coll Cardiol. 2019;74(9):1237–1263. doi: 10.1016/j.jacc.2019.07.012. 31466622 PMC6719727

[16] Patterson T, Rivolo S, Burkhoff D, et al. Physiological impact of afterload reduction on cardiac mechanics and coronary hemodynamics following isosorbide dinitrate administration in ischemic heart disease. J Cardiovasc Transl Res. 2021;14(5):962–974. doi: 10.1007/s12265-021-10112-0. 33721195 PMC8575737

[17] Restivo V, Candiloro S, Daidone M, et al. Systematic review and meta-analysis of cardiovascular risk in rheumatological disease: symptomatic and non-symptomatic events in rheumatoid arthritis and systemic lupus erythematosus. Autoimmun Rev. 2022;21(1):102925. doi: 10.1016/j.autrev.2021.102925. 34454117

[18] Baghdadi LR, Woodman RJ, Shanahan EM, et al. The impact of traditional cardiovascular risk factors on cardiovascular outcomes in patients with rheumatoid arthritis: a systematic review and meta-analysis. PLoS One. 2015;10(2):e0117952. doi: 10.1371/journal.pone.0117952. 25689371 PMC4331556

[19] Santos-Moreno P, Rodriguez-Vargas GS, Martinez S, et al. Metabolic abnormalities, cardiovascular disease, and metabolic syndrome in adult rheumatoid arthritis patients: current perspectives and clinical implications. Open Access Rheumatol. 2022;14:255–267. doi: 10.2147/OARRR.S285407. 36388145 PMC9642585

[20] Kong P, Cui ZY, Huang XF, et al. Inflammation and atherosclerosis: signaling pathways and therapeutic intervention. Signal Transduct Target Ther. 2022;7(1):131. doi: 10.1038/s41392-022-00955-7. 35459215 PMC9033871

[21] Soehnlein O, Libby P. Targeting inflammation in atherosclerosis – from experimental insights to the clinic. Nat Rev Drug Discov. 2021;20(8):589–610. doi: 10.1038/s41573-021-00198-1. 33976384 PMC8112476

[22] Engelen SE, Robinson AJB, Zurke YX, et al. Therapeutic strategies targeting inflammation and immunity in atherosclerosis: how to proceed? Nat Rev Cardiol. 2022;19(8):522–542. doi: 10.1038/s41569-021-00668-4. 35102320 PMC8802279

[23] Pushpakom S, Iorio F, Eyers PA, et al. Drug repurposing: progress, challenges and recommendations. Nat Rev Drug Discov. 2019;18(1):41–58. doi: 10.1038/nrd.2018.168. 30310233

[24] Parvathaneni V, Kulkarni NS, Muth A, et al. Drug repurposing: a promising tool to accelerate the drug discovery process. Drug Discov Today. 2019;24(10):2076–2085. doi: 10.1016/j.drudis.2019.06.014. 31238113 PMC11920972

[25] González L, Bulnes JF, Orellana MP, et al. The role of colchicine in atherosclerosis: from bench to bedside. Pharmaceutics. 2022;14(7):1395. doi: 10.3390/pharmaceutics14071395. 35890291 PMC9323936

[26] Bedoui Y, Guillot X, Sélambarom J, et al. Methotrexate an old drug with new tricks. Int J Mol Sci. 2019;20(20):5023. doi: 10.3390/ijms20205023. 31658782 PMC6834162

[27] Cronstein BN, Aune TM. Methotrexate and its mechanisms of action in inflammatory arthritis. Nat Rev Rheumatol. 2020;16(3):145–154. doi: 10.1038/s41584-020-0373-9. 32066940

[28] Wilsdon TD, Whittle SL, Thynne TR, et al. Methotrexate for psoriatic arthritis. Cochrane Database Syst Rev. 2019;1(1):CD012722. doi: 10.1002/14651858.CD012722.pub2. 30656673 PMC6353064

[29] Xu J, Xiao L, Zhu J, et al. Methotrexate use reduces mortality risk in rheumatoid arthritis: a systematic review and meta-analysis of cohort studies. Semin Arthritis Rheum. 2022;55:152031. doi: 10.1016/j.semarthrit.2022.152031. 35671648

[30] Sun KJ, Liu LL, Hu JH, et al. Methotrexate can prevent cardiovascular events in patients with rheumatoid arthritis: an updated meta-analysis. Medicine (Baltimore). 2021;100(7):e24579. doi: 10.1097/MD.0000000000024579. 33607787 PMC7899830

[31] Roubille C, Richer V, Starnino T, et al. The effects of tumour necrosis factor inhibitors, methotrexate, non-steroidal anti-inflammatory drugs and corticosteroids on cardiovascular events in rheumatoid arthritis, psoriasis and psoriatic arthritis: a systematic review and meta-analysis. Ann Rheum Dis. 2015;74(3):480–489. doi: 10.1136/annrheumdis-2014-206624. 25561362 PMC4345910

[32] Xie F, Chen L, Yun H, et al. Benefits of methotrexate use on cardiovascular disease risk among rheumatoid arthritis patients initiating biologic disease-modifying antirheumatic drugs. J Rheumatol. 2021;48(6):804–812. doi: 10.3899/jrheum.191326. 33060309

[33] Sendaydiego X, Gold LS, Dubreuil M, et al. Comparative safety of biologic and targeted synthetic disease-modifying anti-rheumatic drugs for cardiovascular outcomes in rheumatoid arthritis. Rheumatology (Oxford). 2025;64(6):3434–3443. doi: 10.1093/rheumatology/keaf096. 39936579 PMC12107039

[34] Mangoni AA, Sotgia S, Zinellu A, et al. Methotrexate and cardiovascular prevention: an appraisal of the current evidence. Ther Adv Cardiovasc Dis. 2023;17:17539447231215213. doi: 10.1177/17539447231215213. 38115784 PMC10732001

[35] Baker JF, Sauer B, Teng CC, et al. Initiation of disease-modifying therapies in rheumatoid arthritis is associated with changes in blood pressure. J Clin Rheumatol. 2018;24(4):203–209. doi: 10.1097/RHU.0000000000000736. 29664818 PMC7461421

[36] Mangoni AA, Baghdadi LR, Shanahan EM, et al. Methotrexate, blood pressure and markers of arterial function in patients with rheumatoid arthritis: a repeated cross-sectional study. Ther Adv Musculoskelet Dis. 2017;9(9):213–229. doi: 10.1177/1759720X17719850. 28932292 PMC5600310

[37] Woodman RJ, Baghdadi LR, Shanahan ME, et al. The temporal relationship between arterial stiffening and blood pressure is modified by methotrexate treatment in patients with rheumatoid arthritis. Front Physiol. 2017;8:593. doi: 10.3389/fphys.2017.00593. 28861004 PMC5559508

[38] Hadwen B, Stranges S, Barra L. Risk factors for hypertension in rheumatoid arthritis patients-a systematic review. Autoimmun Rev. 2021;20(4):102786. doi: 10.1016/j.autrev.2021.102786. 33609791

[39] Mangoni AA, Wiese MD, Woodman RJ, et al. Methotrexate, blood pressure and arterial function in rheumatoid arthritis: study protocol. Future Cardiol. 2024;20(13):671–683. doi: 10.1080/14796678.2024.2411167. 39387403 PMC11552479

[40] Aletaha D, Neogi T, Silman AJ, et al. 2010 rheumatoid arthritis classification criteria: an American College of Rheumatology/European League Against Rheumatism collaborative initiative. Ann Rheum Dis. 2010;69(9):1580–1588. doi: 10.1136/ard.2010.138461. 20699241

[41] Aletaha D, Neogi T, Silman AJ, et al. 2010 Rheumatoid arthritis classification criteria: an American College of Rheumatology/European League Against Rheumatism collaborative initiative. Arthritis Rheum. 2010;62(9):2569–2581. doi: 10.1002/art.27584. 20872595

[42] Schulz KF, Altman DG, Moher D. CONSORT 2010 statement: updated guidelines for reporting parallel group randomised trials. BMC Med. 2010;8(1):18. doi: 10.1186/1741-7015-8-18. 20334633 PMC2860339

[43] Morgan SL, Baggott JE, Vaughn WH, et al. Supplementation with folic acid during methotrexate therapy for rheumatoid arthritis. A double-blind, placebo-controlled trial. Ann Intern Med. 1994;121(11):833–841. doi: 10.7326/0003-4819-121-11-199412010-00002. 7978695

[44] Tanaka Y. Subcutaneous injection of methotrexate: advantages in the treatment of rheumatoid arthritis. Mod Rheumatol. 2023;33(4):633–639. doi: 10.1093/mr/roac156. 36525530

[45] Tabit CE, Holbrook M, Shenouda SM, et al. Effect of sulfasalazine on inflammation and endothelial function in patients with established coronary artery disease. Vasc Med. 2012;17(2):101–107. doi: 10.1177/1358863X12440117. 22496207 PMC3632403

[46] Nichols WW, Singh BM. Augmentation index as a measure of peripheral vascular disease state. Curr Opin Cardiol. 2002;17(5):543–551. doi: 10.1097/00001573-200209000-00016. 12357133

[47] van Riel PL, Renskers L. The Disease Activity Score (DAS) and the Disease Activity Score using 28 joint counts (DAS28) in the management of rheumatoid arthritis. Clin Exp Rheumatol. 2016;34(5 Suppl 101):S40–S44. 27762189

[48] Inoue E, Yamanaka H, Hara M, et al. Comparison of Disease Activity Score (DAS)28-erythrocyte sedimentation rate and DAS28-C-reactive protein threshold values. Ann Rheum Dis. 2007;66(3):407–409. doi: 10.1136/ard.2006.054205. 16926186 PMC1856019

[49] Nikiphorou E, Radner H, Chatzidionysiou K, et al. Patient global assessment in measuring disease activity in rheumatoid arthritis: a review of the literature. Arthritis Res Ther. 2016;18(1):251. doi: 10.1186/s13075-016-1151-6. 27793211 PMC5086038

[50] Unger T, Borghi C, Charchar F, et al. 2020 International Society of Hypertension Global Hypertension Practice Guidelines. Hypertension. 2020;75(6):1334–1357. doi: 10.1161/HYPERTENSIONAHA.120.15026. 32370572

[51] Butlin M, Qasem A. Large artery stiffness assessment using SphygmoCor technology. Pulse (Basel). 2017;4(4):180–192. doi: 10.1159/000452448. 28229053 PMC5290450

[52] Marsh S, Collie-Duguid ES, Li T, et al. Ethnic variation in the thymidylate synthase enhancer region polymorphism among Caucasian and Asian populations. Genomics. 1999;58(3):310–312. doi: 10.1006/geno.1999.5833. 10373329

[53] Mauritz R, Giovannetti E, Beumer IJ, et al. Polymorphisms in the enhancer region of the thymidylate synthase gene are associated with thymidylate synthase levels in normal tissues but not in malignant tissues of patients with colorectal cancer. Clin Colorectal Cancer. 2009;8(3):146–154. doi: 10.3816/CCC.2009.n.024. 19632929

[54] Andreson R, Puurand T, Remm M. SNPmasker: automatic masking of SNPs and repeats across eukaryotic genomes. Nucleic Acids Res. 2006;34(Web Server issue):W651–5. doi: 10.1093/nar/gkl125. 16845091 PMC1538889

[55] Phan L, Jin Y, Zhang H, et al. ALFA: allele frequency aggregator. Bethesda, MD: National Center for Biotechnology Information, U.S. National Library of Medicine; 2020. www.ncbi.nlm.nih.gov/snp/docs/gsr/alfa/

[56] Johnson TM, Sayles HR, Baker JF, et al. Investigating changes in disease activity as a mediator of cardiovascular risk reduction with methotrexate use in rheumatoid arthritis. Ann Rheum Dis. 2021;80(11):1385–1392. doi: 10.1136/annrheumdis-2021-220125. 34049859 PMC8516691

[57] Laurent S, Boutouyrie P. The structural factor of hypertension: large and small artery alterations. Circ Res. 2015;116(6):1007–1021. doi: 10.1161/CIRCRESAHA.116.303596. 25767286

[58] Fan Y, Wu L, Zhuang W. Methylenetetrahydrofolate reductase gene rs1801133 and rs1801131 polymorphisms and essential hypertension risk: a comprehensive analysis. Cardiovasc Ther. 2022;2022:2144443–2144418. doi: 10.1155/2022/2144443. 35284002 PMC8888071

[59] Johnson RJ, Kang DH, Feig D, et al. Is there a pathogenetic role for uric acid in hypertension and cardiovascular and renal disease? Hypertension. 2003;41(6):1183–1190. doi: 10.1161/01.HYP.0000069700.62727.C5. 12707287

[60] Zhang L, Spencer KL, Voruganti VS, et al. Association of functional polymorphism rs2231142 (Q141K) in the ABCG2 gene with serum uric acid and gout in 4 US populations: the PAGE Study. Am J Epidemiol. 2013;177(9):923–932. doi: 10.1093/aje/kws330. 23552988 PMC4023295

[61] Blood pressure lowering treatment trialists C. Pharmacological blood pressure lowering for primary and secondary prevention of cardiovascular disease across different levels of blood pressure: an individual participant-level data meta-analysis. Lancet. 2021;397:1625–1636. doi: 10.1016/S0140-6736(21)00590-0. 33933205 PMC8102467

[62] Cui J, Ridker PM, Solomon DH. Blood pressure changes during methotrexate treatment: results from a randomized placebo-controlled trial among patients with cardiovascular risk. Rheumatology (Oxford). 2025;64(6):3917–3920. doi: 10.1093/rheumatology/keae604. 39657237 PMC12107077

[63] Mangoni AA, Sherwood RA, Asonganyi B, et al. Short-term oral folic acid supplementation enhances endothelial function in patients with type 2 diabetes. Am J Hypertens. 2005;18(2 Pt 1):220–226. doi: 10.1016/j.amjhyper.2004.08.036. 15752950

[64] Huo Y, Li J, Qin X, et al. Efficacy of folic acid therapy in primary prevention of stroke among adults with hypertension in China: the CSPPT randomized clinical trial. JAMA. 2015;313(13):1325–1335. doi: 10.1001/jama.2015.2274. 25771069

[65] Asbaghi O, Salehpour S, Rezaei Kelishadi M, et al. Folic acid supplementation and blood pressure: a GRADE-assessed systematic review and dose-response meta-analysis of 41,633 participants. Crit Rev Food Sci Nutr. 2023;63(13):1846–1861. doi: 10.1080/10408398.2021.1968787. 34478339

[66] Mebrahtu TF, Morgan AW, West RM, et al. Oral glucocorticoids and incidence of hypertension in people with chronic inflammatory diseases: a population-based cohort study. CMAJ. 2020;192(12):E295–E301. doi: 10.1503/cmaj.191012. 32392512 PMC7101178

[67] Johnson AG, Nguyen TV, Day RO. Do nonsteroidal anti-inflammatory drugs affect blood pressure? A meta-analysis. Ann Intern Med. 1994;121(4):289–300. doi: 10.7326/0003-4819-121-4-199408150-00011. 8037411

[68] Gomides APM, Albuquerque CP, Santos ABV, et al. High levels of polypharmacy in rheumatoid arthritis-a challenge not covered by current management recommendations: data from a large real-life study. J Pharm Pract. 2021;34(3):365–371. doi: 10.1177/0897190019869158. 31451091

[69] Miyake H, Sada RM, Akebo H, et al. Prevalence and factors associated with polypharmacy among patients with rheumatoid arthritis: a single-centre, cross-sectional study. Clin Rheumatol. 2023;42(9):2287–2295. doi: 10.1007/s10067-023-06646-0. 37243802

