# Supplementary material for: A controlled comparative study of the effects of methotrexate and pharmacogenetic factors on arterial blood pressure and arterial stiffness in patients with rheumatoid arthritis
Source: Ann Med. 2025 Jul 31;57(1):2539311. doi: 10.1080/07853890.2025.2539311 (PMC12315184; doi:10.1080/07853890.2025.2539311)
Supplement: CONSORT_flow_diagram.doc [file IANN_A_2539311_SM7323.doc]

**CONSORT 2010 Flow Diagram**

**Allocation**

**Analysis**

**Follow-Up**

**Enrollment**

Assessed for eligibility (n=144)

Excluded (n=82)

♦  Not meeting inclusion criteria (n=34)

♦  Declined to participate (n=19)

♦  Other reasons (n=29)

Randomized (n=62)

Allocated to intervention, Group 1 (n=31)

♦ Received allocated intervention (n=31)

♦ Did not receive allocated intervention (give reasons) (n=0)

Allocated to intervention, Group 2 (n=31)

♦ Received allocated intervention (n=30)

♦ Did not receive allocated intervention (give reasons) (n=1)*

Lost to follow-up (give reasons) (n=2)^

Discontinued intervention (give reasons) (n=3)^#^

Lost to follow-up (give reasons) (n=4)^

Discontinued intervention (give reasons) (n=11)^#^

Analysed (n=31)
♦ Excluded from analysis (give reasons) (n=0)

Analysed (n=31)
♦ Excluded from analysis (give reasons) (n=0)

**^*^**reason: patient changed their mind and refused to start therapy

^reasons: fail to attend/comply, no longer eligible, or dropped out of study for personal reasons

^#^reasons: adverse events or alternative therapy commenced
