# Supplementary material for: A controlled comparative study of the effects of methotrexate and pharmacogenetic factors on arterial blood pressure and arterial stiffness in patients with rheumatoid arthritis
Source: Ann Med. 2025 Jul 31;57(1):2539311. doi: 10.1080/07853890.2025.2539311 (PMC12315184; doi:10.1080/07853890.2025.2539311)
Supplement: Supplementary_Tables.docx [file IANN_A_2539311_SM7322.docx]

**Supplementary table 1.** Inclusion, exclusion, and withdrawal criteria.

**Inclusion criteria**

- Patients with rheumatoid arthritis diagnosed according to the EULAR/ACR 2010 criteria.
- Age ≥18 years.
- Written informed consent, dated, and signed before initiating any study-related procedure.

**Exclusion criteria**

- Contraindication to methotrexate or sulfasalazine.
- Patient who cannot be followed for six months.
- Active alcohol or substance abuse within the last 12 months.
- Participation in a clinical trial within three months prior to the start of the study.
- Body mass index >35 Kg/m^2^.
- Secondary causes of hypertension.
- Clinic blood pressure >160/100 mmHg.
- Resistant hypertension.
- Clinical systolic blood pressure <100 mmHg or symptomatic orthostatic hypotension.
- Cardiovascular event, procedure, or hospitalization within the last six months.
- Atrial fibrillation.
- Heart failure.
- Treatment with nitrates.
- Estimated glomerular filtration rate (eGFR) <45 mL/min.
- Diagnosis of polycystic kidney disease.
- Glomerulonephritis treated with or likely to be treated with immunosuppressant drugs.
- Uncontrolled diabetes with glycated haemoglobin >9.0% (>75 mmol/mol).
- Uncontrolled dyslipidaemia with total cholesterol >7.5 mmol/L or triglycerides >5.6 mmol/L.
- Clinical diagnosis of dementia, treatment with medications for dementia or, in the opinion of the study staff, the participant is cognitively unable to follow the protocol.
- Other medical, psychiatric, or behavioural factors that may interfere with participation.
- Cancer diagnosed and treated within the past two years that would compromise participation.
- Any organ transplant.
- Pregnancy, currently trying to become pregnant, or of childbearing potential and not using birth control.
- Significant illness within two weeks of study commencement.
- Patients with an unstable active medical condition that could impair participation.

**Withdrawal criteria**

- Treatment-related toxicity.
- Voluntary withdrawal from study by participant.
- Any other intercurrent medical condition or circumstance that precludes completion of the study.

**Supplementary table 2.** Genotypic and allelic frequences in Group 1 patients compared to global frequencies.

| **Group 1** | **Genotype number (% of population)** | | | | **Population allele frequency** | | **Global allele frequency** | |
| --- | --- | --- | --- | --- | --- | --- | --- | --- |
|  | **Reference**  **(R/R)** | **Heterozygous**  **(R/a)** | **Alternate**  **(a/a)** | **Total** | **Reference**  **(R)** | **Alternate**  **(a)** | **Reference**  **(R)** | **Alternate**  **(a)** |
| rs719235 (C→A) | 18 (58.1%) | 11 (35.5%) | 2 (6.4%) | 31 (100%) | 0.758 | 0.242 | 0.747 | 0.252 |
| rs1979277 (G→A) | 11 (35.5%) | 14 (45.1%) | 6 (19.4%) | 31 (100%) | 0.581 | 0.419 | 0.69 | 0.31 |
| rs1801394 (A→G) | 6 (19.4%) | 17 (54.8%) | 8 (25.8%) | 31 (100%) | 0.468 | 0.532 | 0.484 | 0.515 |
| rs1805087 (A→G) | 18 (58.1%) | 12 (38.7%) | 1 (3.2%) | 31 (100%) | 0.774 | 0.226 | 0.808 | 0.192 |
| rs2372536 (C→G) | 16 (51.6%) | 9 (29%) | 6 (19.4%) | 31 (100%) | 0.661 | 0.339 | 0.687 | 0.312 |
| rs17602729 (G→A) | 21 (67.7%) | 10 (32.3%) | 0 (0%) | 31 (100%) | 0.839 | 0.161 | 0.882 | 0.117 |
| rs1801131 (T→G) | 14 (45.1%) | 14 (45.1%) | 3 (9.7%) | 31 (100%) | 0.677 | 0.323 | 0.697 | 0.302 |
| rs2231142 (G→T) | 23 (74.2%) | 7 (22.6%) | 1 (3.2%) | 31 (100%) | 0.855 | 0.145 | 0.899 | 0.101 |
| rs1045642 (A→G) | 6 (19.4%) | 17 (54.8%) | 8 (25.8%) | 31 (100%) | 0.468 | 0.532 | 0.503 | 0.497 |
| rs2273697 (G→A) | 21 (67.7%) | 9 (29%) | 1 (3.2%) | 31 (100%) | 0.823 | 0.177 | 0.801 | 0.199 |
| rs1051266 (T→C) | 12 (38.7%) | 15 (48.4%) | 4 (12.9%) | 31 (100%) | 0.629 | 0.371 | 0.56 | 0.44 |
| rs1801133 (G→A) | 17 (54.8%) | 11 (35.5%) | 3 (9.7%) | 31 (100%) | 0.726 | 0.274 | 0.663 | 0.337 |

**Supplementary table 3.** Genotypic and allelic frequences in Group 2 patients compared to global frequencies.

| **Group 2** | **Genotype number (% of population)** | | | | **Population allele frequency** | | **Global allele frequency** | |
| --- | --- | --- | --- | --- | --- | --- | --- | --- |
|  | **Reference**  **(R/R)** | **Heterozygous**  **(R/a)** | **Alternate**  **(a/a)** | **Total** | **Reference**  **(R)** | **Alternate**  **(a)** | **Reference**  **(R)** | **Alternate**  **(a)** |
| rs719235 (C→A) | 18 (58.1%) | 11 (35.5%) | 2 (6.4%) | 31 (100%) | 0.758 | 0.242 | 0.747 | 0.252 |
| rs1979277 (G→A) | 15 (48.4%) | 11 (35.5%) | 5 (16.1%) | 31 (100%) | 0.661 | 0.339 | 0.69 | 0.31 |
| rs1801394 (A→G) | 5 (16.1%) | 10 (32.3%) | 16 (51.6%) | 31 (100%) | 0.323 | 0.677 | 0.484 | 0.515 |
| rs1805087 (A→G) | 20 (64.5%) | 10 (32.3%) | 1 (3.2%) | 31 (100%) | 0.806 | 0.194 | 0.808 | 0.192 |
| rs2372536 (C→G) | 14 (45.1%) | 13 (41.9%) | 4 (12.9%) | 31 (100%) | 0.661 | 0.339 | 0.687 | 0.312 |
| rs17602729 (G→A) | 26 (83.9%) | 4 (12.9%) | 1 (3.2%) | 31 (100%) | 0.903 | 0.097 | 0.882 | 0.117 |
| rs1801131 (T→G) | 16 (51.6%) | 13 (41.9%) | 2 (6.4%) | 31 (100%) | 0.726 | 0.274 | 0.697 | 0.302 |
| rs2231142 (G→T) | 26 (83.9%) | 4 (12.9%) | 1 (3.2%) | 31 (100%) | 0.903 | 0.097 | 0.899 | 0.101 |
| rs1045642 (A→G) | 5 (16.1%) | 19 (61.3%) | 7 (22.6%) | 31 (100%) | 0.468 | 0.532 | 0.497 | 0.503 |
| rs2273697 (G→A) | 21 (67.7%) | 9 (29%) | 1 (3.2%) | 31 (100%) | 0.823 | 0.177 | 0.801 | 0.199 |
| rs1051266 (T→C) | 7 (22.6%) | 9 (29%) | 15 (48.4%) | 31 (100%) | 0.371 | 0.629 | 0.439 | 0.56 |
| rs1801133 (G→A) | 15 (48.4%) | 11 (35.5%) | 5 (16.1%) | 31 (100%) | 0.661 | 0.339 | 0.663 | 0.337 |

**Supplementary table 4.** ANCOVA analysis of genetic polymorphisms in interaction with sulfasalazine treatment on change in blood pressure and disease activity at one- and six-months follow-up.

1-month sulfasalazine (Group 2)

|  | **rs1801131 (MTHFR)** | | | **rs1051266 (SLC19A1)** | | | **rs1979277 (SHMT)** | | | **rs1045642 (ABCB1)** | | | | | |
| --- | --- | --- | --- | --- | --- | --- | --- | --- | --- | --- | --- | --- | --- | --- | --- |
|  | **TT+TG** | **GG** | **p-value** | **TT** | **TC+CC** | **p-value** | **GG** | **GA+AA** | **p-value** | **AA** | **AG+GG** | **p-value** | **AA+AG** | **GG** | **p-value** |
| **SBP** | -0.28±2.04 | -0.96±7.3 | 0.93 | -6.38±2.65 | 5.53±2.6 | **0.003** | -2.64±2.72 | 2.08±2.76 | 0.228 | 4.58±3.73 | -2.06±2.23 | 0.132 | 0.74±2.12 | -5.94±4.96 | 0.224 |
| **DBP** | -1.73±1.42 | 3.62±5.01 | 0.31 | -4.92±1.96 | 1.95±1.85 | **0.017 *** | -4.74±1.79 | 2.18±1.83 | **0.009 *** | -1.87±2.74 | -1.1±1.6 | 0.808 | -1.52±1.51 | -0.04±3.51 | 0.7 |
| **PP** | 1.21±1.57 | -4.39±5.61 | 0.34 | -1.85±2.08 | 3.55±2.07 | 0.075 | 2.02±2.11 | -0.39±2.16 | 0.429 | 5.98±2.83 | -0.99±1.68 | **0.039** | 2.20±1.62 | -6.08±3.74 | **0.05 *** |
| **DAS28-CRP** | -0.74±0.2 | 0.86±0.7 | **0.033 *** | -0.89±0.31 | -0.41±0.28 | 0.28 | -0.8±0.28 | -0.42±0.3 | 0.35 | -0.99±0.43 | -0.51±0.23 | 0.317 | -0.74±0.22 | -0.01±0.51 | 0.182 |

|  | **rs2372536 (ATIC)** | | | **TS 28bp** | | |
| --- | --- | --- | --- | --- | --- | --- |
|  | **CC+GG** | **GG** | **p-value** | **2R/2R + 2R/3R** | **3R/3R** | **p-value** |
| **SBP** | -1.21±2.11 | 4.52±4.95 | 0.293 | 0.52±2.53 | -0.98±3.32 | 0.728 |
| **DBP** | -2.44±1.42 | 5.30±3.39 | **0.04** | -0.63±1.83 | -2.51±2.35 | 0.537 |
| **PP** | 1.22±1.65 | -1.46±4.02 | 0.541 | 0.86±1.94 | 1.61±2.56 | 0.818 |
| **DAS28-CRP** | -0.54±0.22 | -1.05±0.51 | 0.353 | -0.35±0.26 | -1.20±0.32 | **0.049 *** |

6-month sulfasalazine (Group 2)

|  | **rs1801131 (MTHFR)** | | | **rs2273697 (ABCC2)** | | | **rs719235 (GGH)** | | | **rs1051266 (SLC19A1)** | | | **TS 28bp** | | |
| --- | --- | --- | --- | --- | --- | --- | --- | --- | --- | --- | --- | --- | --- | --- | --- |
|  | **TT** | **TG+GG** | **p-value** | **GG** | **GA+AA** | **p-value** | **CC** | **CA+AA** | **p-value** | **TT** | **TC+CC** | **p-value** | **2R/2R**  **+2R/3R** | **3R/3R** | **p-value** |
| **SBP** | 5.79±3.13 | -4.98±3.36 | **0.027** | -4.44±3.22 | 6.73±3.47 | **0.025 *** | 0.28±3.27 | 1.46±4.01 | 0.825 | -3.75±3.57 | 4.66±3.31 | 0.105 | -2.22±3.21 | 5.25±3.98 | 0.167 |
| **DBP** | 4.09±2.28 | -2.63±2.54 | 0.06 | -1.3±2.44 | 3.57±2.56 | 0.182 | -2.12±2.22 | 5.17±2.62 | **0.047** | -3.86±2.59 | 4.85±2.28 | **0.02** | 1.68±2.35 | 0.16±2.84 | 0.687 |
| **PP** | 1.57±2.68 | -2.63±2.84 | 0.289 | -3.35±2.67 | 3.05±2.85 | 0.111 | 1.74±2.55 | -3.46±3.1 | 0.205 | -0.19±2.79 | -0.5±2.67 | 0.937 | -3.75±2.44 | 5.03±3.13 | **0.037 *** |
| **DAS28-CRP** | -0.34±0.45 | -1.14±0.45 | 0.254 | -0.84±0.42 | -0.65±0.5 | 0.772 | -0.53±0.44 | -1.06±0.49 | 0.428 | -0.66±0.47 | -0.86±0.46 | 0.763 | -0.33±0.42 | -1.35±0.49 | 0.127 |

Legend: SBP, systolic blood pressure; DBP, diastolic blood pressure; PP, pulse pressure; DAS28-CRP, disease activity score-28, MTHFR, methyl tetrahydrofolate reductase; SLC19A1, Solute carrier family 19 member 1; SHMT, Serine hydroxymethyltransferase 1; ABCB1, ATP-binding cassette subfamily B member 1; ATIC, 5-aminoimidazole-4-carboxamide ribonucleotide formyltransferase; TS 28bp, thymidylate synthase promoter 28bp repeat; ABCC2, ATP-binding cassette subfamily C member 2; GGH, Gamma-glutamyl hydrolase.

Values are estimated means±SE. All adjusted for age, sex and baseline measurement of dependent variable. SBP, DBP and PP are expressed as change in mmHg.

*: Not significant at ANCOVA level, only at pairwise comparison.
